# Unusual Hemodialysis Catheter Trajectory in a Patient with ESKD

**DOI:** 10.34067/KID.0000000831

**Published:** 2025-11-20

**Authors:** Luis Rafael Alvarez Velazquez, Sara Isabel Estrada Intriago

**Affiliations:** Mexican Social Security Institute (Instituto Mexicano del Seguro Social), Morelia, Mexico

**Keywords:** chronic hemodialysis, hemodialysis, hemodialysis access, vascular access

## Abstract

**Figure:**
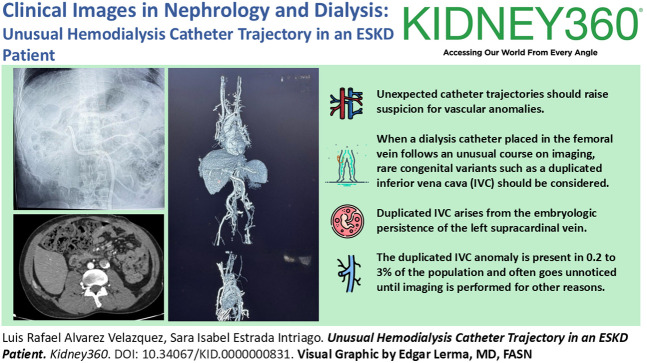

## Case Description

A 28-year-old man with a history of posterior urethral valves corrected in the neonatal period developed ESKD, received a kidney transplant in 2002, and resumed hemodialysis in 2016 because of chronic graft dysfunction. He had a history of syndactyly, hypertension, multiple thrombosed vascular accesses, and severe superior vena cava (SVC) stenosis without clinical signs of SVC syndrome.

**Figure 1:**
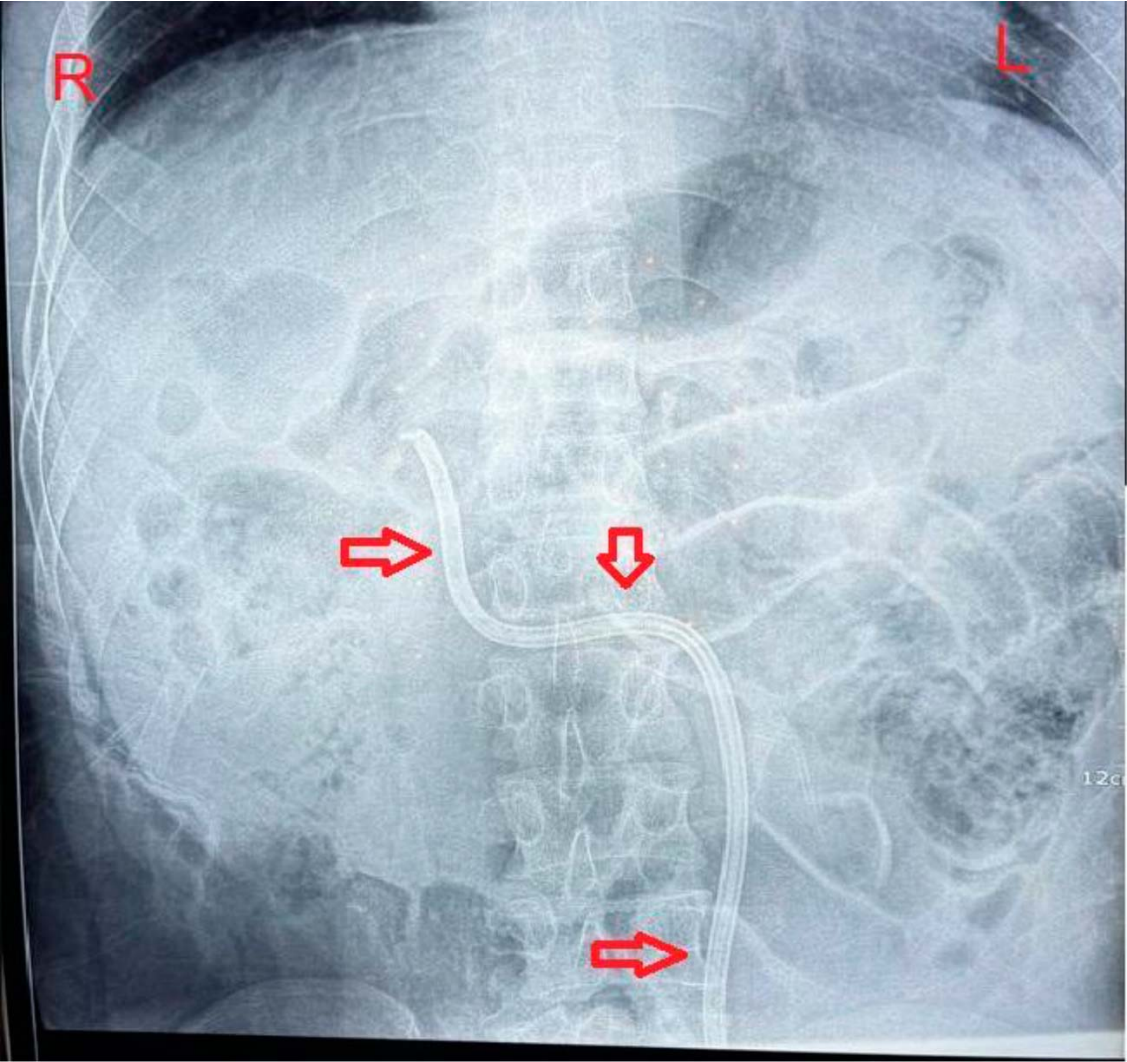
**Abdominal radiograph of a tunneled venous catheter shows an atypical, serpiginous subcutaneous course ascending from the left inguinal region toward the upper abdomen.** The catheter's tip is directed toward the right upper quadrant, likely terminating near the right suprahepatic vein. Red arrows highlight the path of the catheter along its intravascular trajectory.

**Figure 2:**
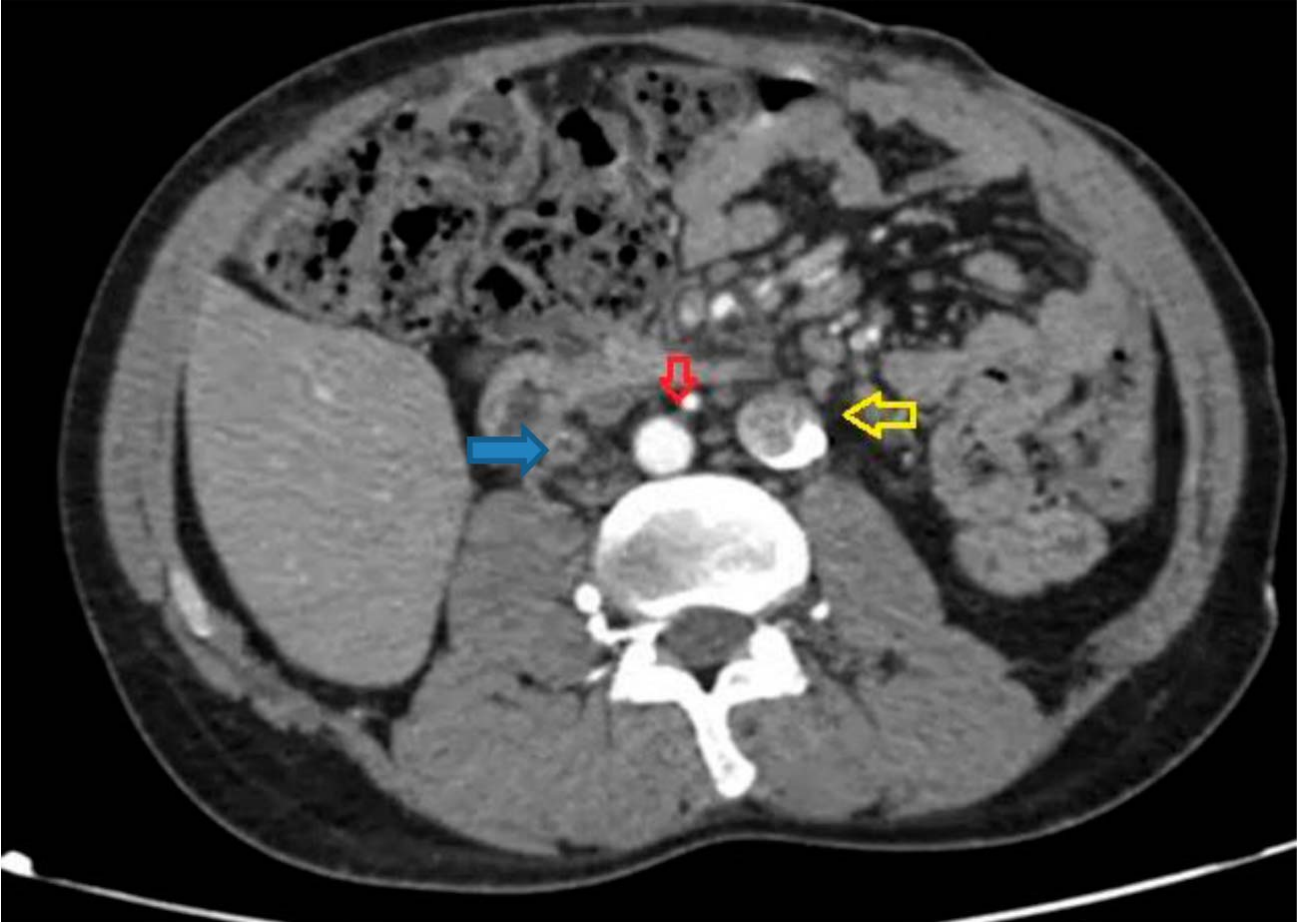
**Axial CT scan image demonstrates two venous structures of significant caliber running parallel on both sides of the abdominal aorta, consistent with a duplicated IVC.** Both vessels converge at the level of the renal veins, confirming the presence of IVC duplication. The red arrow points to the abdominal aorta, the yellow arrow indicates the left-sided duplicated IVC containing the tunneled catheter, and the blue arrow shows the right-sided IVC, which appears reduced in caliber. CT, computed tomography; IVC, inferior vena cava.

**Figure 3:**
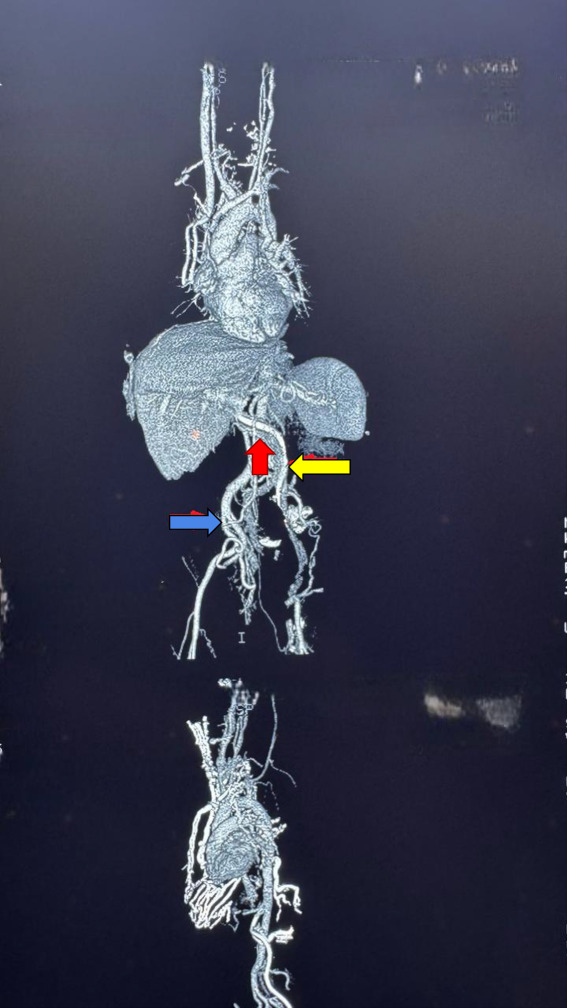
**3D CT angiographic reconstruction demonstrates duplication of the IVC, with two parallel ascending venous pathways originating from the lower extremities.** These vessels converge at the level of the renal veins—highlighted by the red arrow—forming a single IVC above this point. The yellow arrow indicates the left-sided IVC and the intravascular trajectory of the catheter. By contrast, the blue arrow points to the right-sided IVC, which shows a markedly reduced caliber compared with the contralateral vein, consistent with significant stenosis. In addition, there is evidence of severe stenosis of the SVC, characterized by pronounced narrowing of its lumen in the upper thoracic segment. Associated collateral circulation is visible, suggestive of compensatory venous hypertension. SVC, superior vena cava.

After dysfunction of a tunneled right femoral dual-lumen dialysis catheter, a new catheter was inserted into the left femoral vein. A plain abdominal radiograph revealed a serpiginous course of the catheter (Figure 1). Subsequent axial contrast-enhanced computed tomography demonstrated a duplicated inferior vena cava (IVC) with convergence at the renal level and severe stenosis of the SVC (Figure 2). A 3D computed tomography reconstruction confirmed the catheter termination in the right suprahepatic vein and delineated the venous anomaly (Figure 3). The catheter remains functional; however, the patient is under close surveillance given his elevated thrombotic risk.

## Discussion

Duplicated IVC is a rare congenital anomaly, occurring in approximately 0.2% to 3% of the general population. It results from the persistence of the left supracardinal vein during embryologic development.^1^ Although usually clinically asymptomatic, this anomaly may be incidentally discovered during imaging or invasive procedures. It has clinical implications during placement of femoral vein catheters, IVC filters, or retroperitoneal surgeries.^2,3^

In our case, the anomalous catheter trajectory on fluoroscopy prompted further imaging, revealing a duplicated IVC. Recognition of this variant prevented unnecessary repositioning and ensured appropriate catheter function. Familiarity with vascular anomalies, such as duplicated IVC, is essential in nephrology and interventional procedures to avoid misinterpretation and complications.^4^

## Teaching Point

- Unexpected catheter trajectories should raise suspicion for vascular anomalies.
- When a dialysis catheter placed in the femoral vein follows an unusual course on imaging, rare congenital variants, such as a duplicated IVC, should be considered.
- Duplicated IVC arises from the embryologic persistence of the left supracardinal vein.
- The duplicated IVC anomaly is present in 0.2%–3% of the population and often goes unnoticed until imaging is performed for other reasons.
